# Development and validation of an immune-based nomogram model for predicting severe adenovirus pneumonia in hospitalized children

**DOI:** 10.3389/fped.2026.1742222

**Published:** 2026-03-11

**Authors:** Yuting Wu, Xiaolin Ma, Wenquan Niu, Ling Cao

**Affiliations:** 1 Department of Respiratory Medicine, Capital Center for Children’s Health, Capital Institute of Pediatrics, Capital Medical University, Beijing, China; 2 Center for Evidence-Based Medicine, Capital Center for Children’s Health, Capital Institute of Pediatrics, Capital Medical University, Beijing, China

**Keywords:** children, human adenovirus, immune, nomogram, prediction, severe pneumonia

## Abstract

**Background:**

Human adenovirus (HAdV) is a significant cause of severe pneumonia in children that often causes sequelae. Although immune disorders are known to be associated with disease progression, comprehensive immunological predictors have not been identified. The purpose of this study was to explore the ability of multiple immunological indicators to predict severe adenovirus pneumonia (SAP) and to develop an immune-based nomogram for the early prediction of SAP in children.

**Methods:**

This study involved a retrospective analysis of children with adenovirus pneumonia who were hospitalized and received treatment at the Department of Respiratory Medicine, Capital Center for Children's Health, Capital Medical University between January 2017 and June 2025. Patients were stratified into mild and severe groups on the basis of clinical manifestations. They were subsequently randomly allocated at an 80:20 ratio into a training set and a cross-validation set for nomogram development and validation. R software (version 4.4.3) was used for statistical analysis, and effect sizes are expressed as odds ratios (ORs) and 95% confidence intervals (CIs).

**Results:**

Among the 1220 cases included, 357 (29.3%) were classified as severe. In both the training and cross-validation sets, patients with SAP were younger and had longer hospital stays (all *P* < 0.001). After the adjustments for age and sex, logistic regression in the training set revealed seven significant factors associated with SAP occurrence in children: *Mycoplasma pneumoniae* infection (OR = 1.372, 95% CI: 1.04–1.809, *P* < 0.001); complement component 3 (C3) (OR = 0.234, 95% CI: 0.132–0.417, *P* < 0.001) and 4 (C4) (OR = 0.075, 95% CI: 0.018–0.31, *P* < 0.001); immunoglobulin G (IgG) (OR = 1.049, 95% CI: 1.028–1.071, *P* < 0.001); and the percentages of CD3^+^ [CD3^+^ (%)] (OR = 0.965, 95% CI: 0.952–0.979, *P* < 0.001), CD4^+^ [CD4^+^ (%)] (OR = 0.950, 95% CI: 0.934–0.967, *P* < 0.001), and CD19^+^ cells [CD19^+^ (%)] (OR = 1.042, 95% CI: 1.028–1.057, *P* < 0.001). Furthermore, logistic regression of the validation set revealed C3, C4, IgG, CD3^+^(%), CD4^+^ (%) and CD19^+^(%) as consistent predictors of SAP across datasets. Incorporating the significant factors improved model discrimination, increasing the area under the curve (AUC) from 0.627 to 0.836 in the training set and from 0.678 to 0.913 in the cross-validation set. The final nomogram model based on the significant factors demonstrated strong calibration and discrimination (C-index: 0.731 in the training set, 0.812 in the cross-validation set), supporting its potential for clinical risk stratification.

**Conclusion:**

This study identified and validated seven independent factors significantly associated with SAP in children. A nomogram incorporating these factors was developed and it demonstrated favourable discriminative performance and good calibration. The model exhibited high sensitivity and maintained high predictive accuracy across both datasets, indicating its potential clinical utility for individualized risk stratification.

## 1 Introduction

Human adenovirus (HAdV) causes adenovirus pneumonia (AVP) and is a major aetiological agent of community-acquired pneumonia in children. Owing to the incomplete development of the immune system in children and the lack of specific antiviral drugs, it often causes severe adenovirus pneumonia (SAP), a condition associated with considerable morbidity and potential long-term sequelae (1, 2). In the past decade, researchers have focused on identifying biomarkers and significant factors that can predict SAP severity and prognosis (3–5). Many studies have confirmed that traditional inflammatory indicators, such as the white blood cell count, the neutrophil ratio and lactate dehydrogenase (LDH) levels, are significantly elevated in children with SAP and are associated with disease severity (6, 7). The progression and severity of HAdV infection are closely related to the host immune response. Previous studies have started delineatng the complex immunological profile of SAP. The humoral immune system is strongly activated in SAP, with multiple studies consistently documenting elevated serum levels of immunoglobulin G (IgG), IgA, and IgM, reflecting a vigorous antibody-mediated response to viral challenge (8). Serum levels of various proinflammatory cytokines, such as interleukin-6, tumor necrosis factor-α, interferon-γ, are significantly higher in children with SAP than in those with nonsevere infection (9). Concurrent analysis of cellular immunity also revealed dysregulation. For example, the monocyte count is negatively associated with SAP (10). Moreover, other studies have reported pronounced decreases in CD3^+^ T cells and increases in natural killer(NK) cells, particularly in infections caused by highly virulent serotypes such as HAdV-7 (11).

Despite these advances, key knowledge gaps still exist, and most evidence is still at the descriptive level and lacks integrated analysis (8, 12, 13). The prognostic role of humoral immune components, particularly complement components and immunoglobulins, in SAP is unclear because existing studies have neither determined their significant correlation with severity nor evaluated their combined predictive value. In addition, the current prediction model lacks key cellular immune parameters and relies on nonspecific clinical and biochemical markers. This fragmentation of evidence highlights the need to integrate multiple types of immunological data for systematic investigation.

Therefore, to address this knowledge gap and provide more reference for future research and clinical management, we retrospectively analysed the humoral and cellular immune profiles of a large cohort of children with AVP. This study aimed to identify immunological factors that can predict SAP occurrence and to develop a reliable prediction nomogram model for the early identification of patients who may develop severe disease, thereby guiding timely interventions and improving clinical outcomes.

## 2 Methods

### 2.1 Study design and patients

In this retrospective case–control study, the research subjects were children with AVP who were hospitalized and received treatment at the Department of Respiratory Medicine, Capital Center for Children's Health, Capital Medical University between January 2017 and June 2025. Patients were included in the study if they were younger than 18 years and fulfilled the diagnostic criteria for AVP (14, 15). Patients were excluded if they had chronic lung diseases, tuberculosis, immune system disorders, chromosomal diseases, neuromuscular diseases, inherited metabolic diseases, myelosuppression with hematologic malignancies, cardiovascular system diseases and tumors.

### 2.2 Diagnostic criteria for AVP

Patients who met the following two conditions were diagnosed with AVP: (1) met the diagnostic criteria for community-acquired pneumonia (16) and (2) were positive for HAdV nucleic acid in nasopharyngeal swabs, sputum or bronchoalveolar lavage fluid.

### 2.3 Diagnostic criteria for severe pneumonia

The criteria for severe pneumonia according to the guidelines of the American Thoracic Society for the management of community-acquired pneumonia were as follows (17): (1) Major criteria: invasive mechanical ventilation, fluid refractory shock, acute need for noninvasive positive pressure ventilation (NIPPV), and hypoxemia requiring a fraction of inspired oxygen (FiO2) greater than the inspired concentration or flow feasible in the general care area; (2) Minor criteria: minimum respiratory rate greater than the WHO classification for age, apnoea, increased work of breathing, PaO2/FiO2 ratio < 250, multilobar infiltrates, Pediatric Early Warning Score(PEWS) > 6, altered mental status, hypotension, presence of effusion and unexplained metabolic acidosis. For children with AVP who meet one main or two secondary standards, SAP should be considered.

### 2.4 Data collection

Basic data such as admission data, physical examination at admission, discharge diagnosis and hospitalization days, and combined infection and laboratory examination, were collected. Basic information included sex, age and underlying diseases. Data on comorbid infections involved pathogen nucleic acid detection for organisms such as *Mycoplasma pneumoniae* (MP), the influenza virus and bacteria. Laboratory investigations included measurements of complement components (C3 and C4); immunoglobulins (IgA, IgG and IgM), and percentages of lymphocyte subpopulations such as CD3^+^ T lymphocytes (CD3^+^), CD4^+^ T lymphocytes, CD8^+^ T lymphocytes, B lymphocytes (CD19^+^), and NK cells (CD16^+^/56^+^). In this study, all blood samples used for detecting immunological indicators, including complement components, immunoglobulins, and lymphocyte subsets, were collected in the early morning of the second day after admission.

### 2.5 Statistical analysis

According to the diagnostic criteria of the disease, the children enrolled in the study were divided into a severe group (SAP) or a mild group (MAP). They were subsequently stratified and randomly assigned to a training set (80%) and a cross-validation set (20%) for the development and validation of the nomogram. Continuous data are presented as medians (interquartile ranges) or means (standard deviations), on the basis of their distribution. The Mann–Whitney U test was used for nonnormally distributed data, while the Student's *t*-test was applied for normally distributed data. Categorical data are expressed as numbers (percentages) and were compared using the chi-square (*χ*^2^) test or Fisher's exact test, as appropriate.

Univariate logistic regression was performed to identify factors significantly associated with SAP. Variables that showed significant associations in the univariate analysis were then included in a multivariate logistic regression model, adjusted for age and sex, to determine their independent effects. The results are presented as odds ratios (ORs) with corresponding 95% confidence intervals (95% CIs).

The predictive performance gained by adding the aforementioned identified significant factors to the baseline model was evaluated from both the calibration and discrimination aspects. Correlation analysis was conducted to quantify the collinearity among the study factors. With respect to calibration, the Akaike information criterion (AIC), Bayesian information criterion (BIC) and Hosmer–Lemeshow (HL) test were employed to evaluate the overall goodness-of-fit between the predicted probabilities and the reflectance of both the actual observed risk and the revised risk model, with the incorporation of the identified significant factors (18, 19). Lower AIC and BIC values indicate a better model fit. From the discrimination perspective, the net reclassification improvement (NRI), integrated discrimination improvement (IDI), change in area under the curve (ΔAUC), and decision curve analysis (DCA) were used to evaluate the clinical benefits and utility of the full model relative to the baseline model (20–22). By using DCA, the addition of significant factors clearly demonstrated net benefits compared with the basic model. The net benefit increases with increasing distance from both the horizontal line, which represents mild adenovirus pneumonia (MAP) and the solid curve, which represents all SAP patients. Finally, a nomogram model for predicting SAP occurrence was constructed, and the area under the receiver operating characteristic curve (C-index) was used as an indicator to evaluate the prediction accuracy of the model.

All the statistical analyses were performed using R software (version 4.4.3). A two-sided p value of less than 0.05 was considered to indicate statistical significance.

## 3 Results

### 3.1 Baseline characteristics

**Figure 1:**
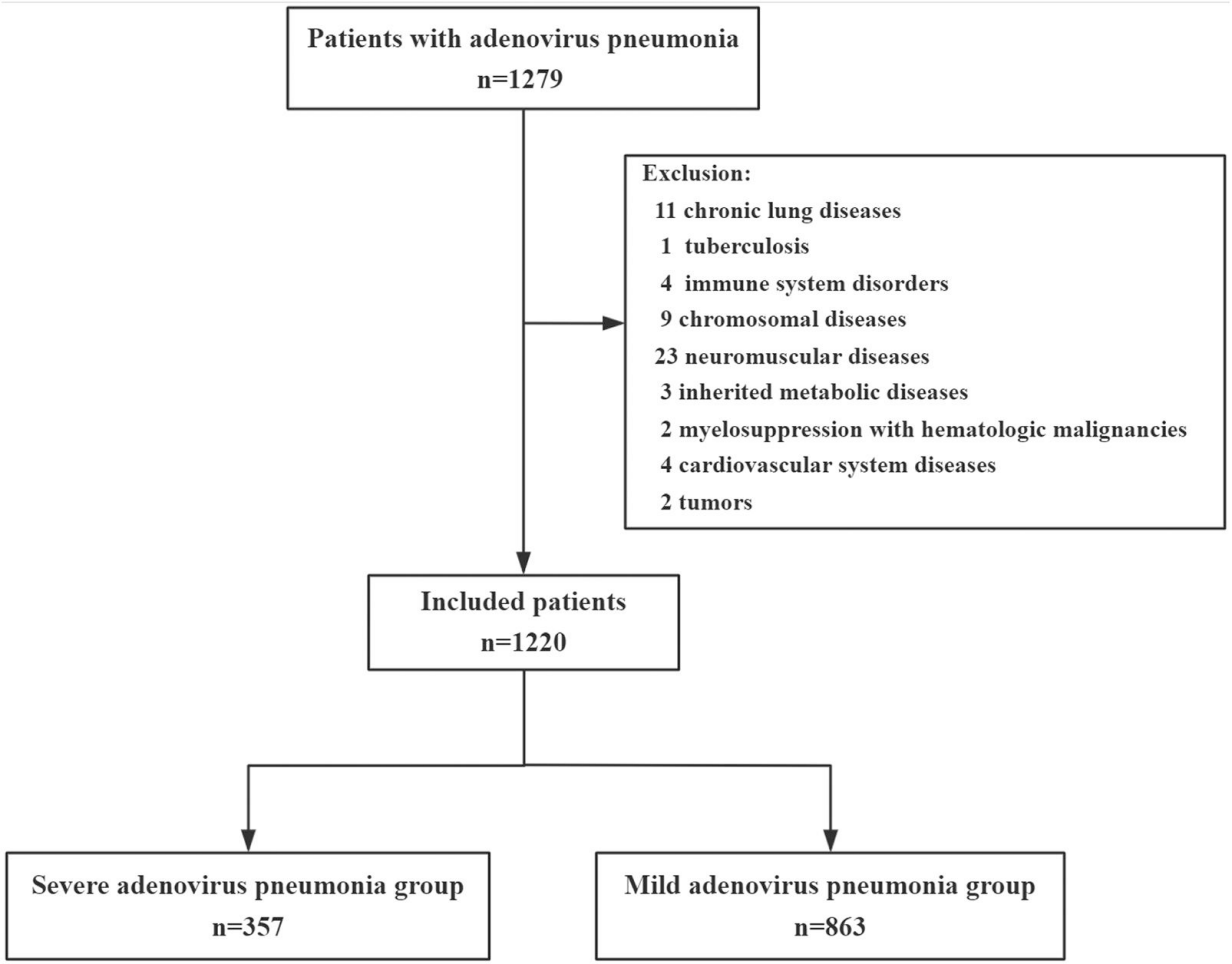
Flow chart of the enrolled participants.

Among the 1279 children diagnosed with AVP in this study, 59 were excluded for the following reasons: chronic lung diseases (*n* = 11), tuberculosis (*n* = 1), immune system disorders (*n* = 4), chromosomal diseases (*n* = 9), neuromuscular diseases (*n* = 23), inherited metabolic diseases (*n* = 3), myelosuppression with hematologic malignancies (*n* = 2), cardiovascular system diseases (*n* = 4) and tumors (*n* = 2). Ultimately, 1220 children with AVP were analysed, including 357 (29.26%) with SAP and 863 (70.74%) with MVP (Figure 1). All 1220 children included in the study were subsequently randomly assigned to the training set or the cross-validation set at a ratio of 80:20 for the development and validation of the nomogram. The baseline characteristics of these patients are provided in Table 1. Notably, in the training set, compared with those in the MAP group, the patients in the SAP group were significantly younger (*P* < 0.001), and the prevalence of MP infection was greater in the SAP group (51.6%) than in the MAP group (43.7%) (*P* = 0.03). Furthermore, the length of hospital stay was significantly longer in the SAP group (*P* < 0.001). A similar pattern was observed in the cross-validation set, where patients in the SAP group were significantly younger and had longer hospital stays compared with patients in the MAP group (both *P* < 0.001). However, in this validation subset, the difference in the prevalence of MP infection between the SAP and MAP groups was not statistically significant.

**Table 1:** Baseline characteristics of study patients.

Set	Training set			Cross-validation set		
Characteristics	Mild AVP (MAP) *n* = 691	Severe AVP (SAP) *n* = 285	*P*	Mild AVP (MAP) *n* = 172	Severe AVP (SAP) *n* = 72	*P*
Age (years)	4.72 [3.00, 7.52]	3.71 [2.16, 6.08]	<0.001	4.42 [2.98, 7.29]	2.71 [1.70, 5.15]	<0.001
Sex (%)			0.086			0.153
Female	290 (42.0)	102 (35.8)		76 (44.2)	24 (33.3)	
Male	401 (58.0)	183 (64.2)		96 (55.8)	48 (66.7)	
Hospital days	5.00 [3.00, 7.00]	8.00 [6.00, 11.00]	<0.001	5.00 [3.00, 6.00]	8.00 [6.00, 12.25]	<0.001
Multi-infection (%)	180 (26.0)	83 (29.1)	0.366	47 (27.3)	19 (26.4)	1
Flu (%)	109 (15.8)	43 (15.1)	0.864	36 (20.9)	8 (11.1)	0.102
MP (%)	302 (43.7)	147 (51.6)	0.03	68 (39.5)	35 (48.6)	0.243
Bacteria (%)	331 (47.9)	145 (50.9)	0.438	89 (51.7)	40 (55.6)	0.687
C3 (g/L)	1.16 [1.03, 1.30]	1.09 [0.89, 1.25]	<0.001	1.20 (0.25)	1.02 (0.25)	<0.001
C4 (g/L)	0.27 [0.20, 0.34]	0.24 [0.17, 0.31]	<0.001	0.29 (0.10)	0.23 (0.09)	<0.001
IgA (g/L)	1.23 [0.67, 1.81]	0.98 [0.54, 1.42]	<0.001	1.26 [0.72, 1.71]	0.67 [0.34, 1.23]	<0.001
IgG (g/L)	9.73 [7.46, 12.15]	9.96 [7.62, 13.80]	0.054	9.64 [8.04, 11.70]	10.15 [8.09, 14.52]	0.069
IgM (g/L)	1.49 [1.05, 2.21]	1.60 [1.02, 2.46]	0.255	1.37 [1.04, 2.16]	1.27 [0.99, 2.02]	0.326
CD3^+^ (%)	67.00 [60.50, 72.00]	63.00 [54.00, 71.00]	<0.001	67.00 [59.00, 73.00]	59.00 [51.00, 67.75]	<0.001
CD4^+^ (%)	36.00 [30.00, 41.00]	31.00 [25.00, 39.00]	<0.001	36.00 [30.00, 42.00]	31.50 [23.75, 39.25]	<0.001
CD8^+^ (%)	25.00 [21.00, 29.98]	25.00 [21.00, 30.00]	0.821	25.00 [21.00, 29.00]	24.00 [19.00, 28.00]	0.065
CD19^+^ (%)	21.00 [16.76, 27.18]	25.00 [18.69, 34.69]	<0.001	21.00 [15.93, 28.48]	30.03 [21.00, 40.57]	<0.001
CD16^+^/56^+^ (%)	10.00 [6.00, 13.00]	8.00 [5.00, 12.00]	0.017	9.00 [6.00, 13.25]	7.00 [4.00, 11.25]	0.025

Hospital days, the number of days the patient was hospitalized; Multi-infections, multiple infections; Flu, influenza infection; MP, *Mycoplasma pneumoniae* infection; Bacteria, bacterial infection; C3, complement component 3; C4, complement component 4; IgA, immunoglobulin A; IgG, immunoglobulin G; IgM, immunoglobulin M; CD3^+^(%), percentage of CD3^+^ T lymphocytes; CD4^+^(%), percentage of CD4^+^ T lymphocytes; CD8^+^(%), percentage of CD8^+^ T lymphocytes; CD19^+^(%), percentage of B lymphocytes; CD16^+^/56^+^ (%), percentage of natural killer cells.

### 3.2 Identification of significant factors for SAP

To explore the critical factors contributing to SAP, we conducted a logistic regression analysis of the relevant indicators. Table 2 presents the results of the analyses for the training set before and after adjustment. In the univariable analysis, MP infection, complement components 3 (C3) and C4, IgA, IgG, and lymphocyte subsets, including percentages of CD3^+^ T lymphocytes [CD3^+^(%)], CD4^+^(%) and CD19^+^(%), were identified as significant predictors (all *P* < 0.05). Following multivariable adjustment, MP infection (OR = 1.372, 95% CI: 1.04–1.809, *P* < 0.001), C3 (OR = 0.234, 95% CI: 0.132–0.417, *P* < 0.001), C4 (OR = 0.075, 95% CI: 0.018–0.31, *P* < 0.001), IgG (OR = 1.049, 95% CI: 1.028–1.071, *P* < 0.001), CD3^+^ (%) (OR = 0.965, 95% CI: 0.952–0.979, *P* < 0.001), CD4^+^ (%) (OR = 0.950, 95% CI: 0.934–0.967, *P* < 0.001) and CD19^+^ (%) (OR = 1.042, 95% CI: 1.028–1.057, *P* < 0.001) remained statistically significant, whereas IgA did not retain significance.

**Table 2:** Identification of significant factors for SAP before and after adjustment in the training set.

Variables	Unadjusted model			Adjusted model		
	OR	95% CI	*P*	OR	95% CI	*P*
MP	1.372	1.04–1.809	0.025	1.801	1.338–2.424	<0.001
C3	0.193	0.109–0.342	<0.001	0.234	0.132–0.417	<0.001
C4	0.081	0.02–0.324	<0.001	0.075	0.018–0.31	<0.001
IgA	0.63	0.517–0.767	<0.001	0.787	0.61–1.015	0.065
IgG	1.039	1.018–1.06	<0.001	1.049	1.028–1.071	<0.001
CD3^+^ (%)	0.960	0.947–0.973	<0.001	0.965	0.952–0.979	<0.001
CD4^+^ (%)	0.953	0.937–0.969	<0.001	0.950	0.934–0.967	<0.001
CD19^+^ (%)	1.049	1.035–1.064	<0.001	1.042	1.028–1.057	<0.001

OR, odds ratio; 95% CI, 95% confidence interval; MP, *Mycoplasma pneumoniae* infection; C3, complement component 3; C4, complement component 4; IgA, immunoglobulin A; IgG, immunoglobulin G; CD3^+^(%), percentage of CD3^+^ T lymphocytes; CD4^+^(%), percentage of CD4^+^ T lymphocytes; CD19^+^(%), percentage of B lymphocytes.

Parallel analyses for the cross-validation set are presented in Table 3. Univariable analysis of this subset revealed that C3 levels, C4 levels, IgA levels, IgG levels, and the CD3^+^ (%), CD4^+^ (%), CD8^+^ (%) and CD19^+^ (%) lymphocyte subsets were significant factors (all *P* < 0.05). After adjustment for age and sex, C3, C4, IgA, IgG, CD3^+^ (%), CD4^+^ (%) and CD19^+^ (%) continued to demonstrate statistical significance, whereas CD8^+^ (%) was not independently significant in the adjusted model. The consistent significance of complement components (C3, C4), IgG and the CD3^+^ (%), CD4^+^ (%) and CD19^+^ (%) lymphocyte subsets in the two datasets before and after adjustment support the internal validity and stability of the important factors related to SAP.

**Table 3:** Identification of significant factors for SAP before and after adjustment in the cross-validation set.

Variables	Unadjusted model			Adjusted model		
	OR	95% CI	*P*	OR	95% CI	*P*
C3	0.047	0.013–0.168	<0.001	0.066	0.018–0.24	<0.001
C4	0.001	0–0.021	<0.001	0.001	0–0.020	<0.001
IgA	0.433	0.278–0.675	<0.001	0.545	0.316–0.938	0.028
IgG	1.072	1.020–1.127	0.006	1.092	1.036–1.152	<0.001
CD3^+^ (%)	0.940	0.915–0.965	<0.001	0.945	0.919–0.971	<0.001
CD4^+^ (%)	0.949	0.921–0.979	0.001	0.953	0.923–0.983	0.003
CD8^+^ (%)	0.953	0.912–0.995	0.03	0.963	0.921–1.006	0.094
CD19^+^ (%)	1.079	1.049–1.110	<0.001	1.072	1.041–1.105	<0.001

OR, odds ratio; 95% CI, 95% confidence interval; C3, complement component 3; C4, complement component 4; IgA, immunoglobulin A; IgG, immunoglobulin G; CD3^+^(%), percentage of CD3^+^ T lymphocytes; CD4^+^ (%), percentage of CD4^+^ T lymphocytes; CD8^+^ (%), percentage of CD8^+^ T lymphocytes; CD19^+^ (%), percentage of B lymphocytes.

### 3.3 Predictive accuracy of independent significant factors

Two models were constructed to assess the prediction performance of the two significant factors identified: the basic model and the full model. In the training set, the basic model includes age, sex, the number of days the patient was hospitalized, influenza infection, bacterial infection, multiple infections, IgA, IgM, and percentages of CD8^+^ T lymphocytes and NK cells. The full model additionally includes the seven significant factors identified in the training set. In the cross-validation set, the basic model includes age, sex, the number of days the patient was hospitalized, influenza infection status, bacterial infection status, MP infection status, multiple infections, IgM status, and percentages of CD8^+^ T lymphocytes and NK cells. The full model additionally includes the seven significant factors in the cross-validation set. Prior to model construction, comprehensive statistical measures were computed to assess predictive accuracy in terms of both calibration and discrimination.

As summarized in Table 4, model fit indices such as the AIC and BIC, along with the HL test (p value), were evaluated in both training and cross-validation sets. Additionally, reclassification metrics, including the NRI and IDI, were calculated.

**Table 4:** Predictive accuracy in models with and without the significant factors when predicting SAP.

Set	Training set		Cross-validation set	
Statistics	Basic model	Full model	Basic model	Full model
Calibration				
AIC	1156.207	916.683	291.936	197.657
BIC	1200.158	1004.585	319.914	260.606
HL test (p)	0.921	0.272	0.678	0.234
Discrimination				
NRI	0.689		0.702	
IDI	0.259		0.381	

AIC, akaike information criterion; BIC, bayesian information criteria; HL test, hosmer-lemeshow test; NRI, net reclassification improvement; IDI, integrated discrimination improvement.

**Figure 2:**
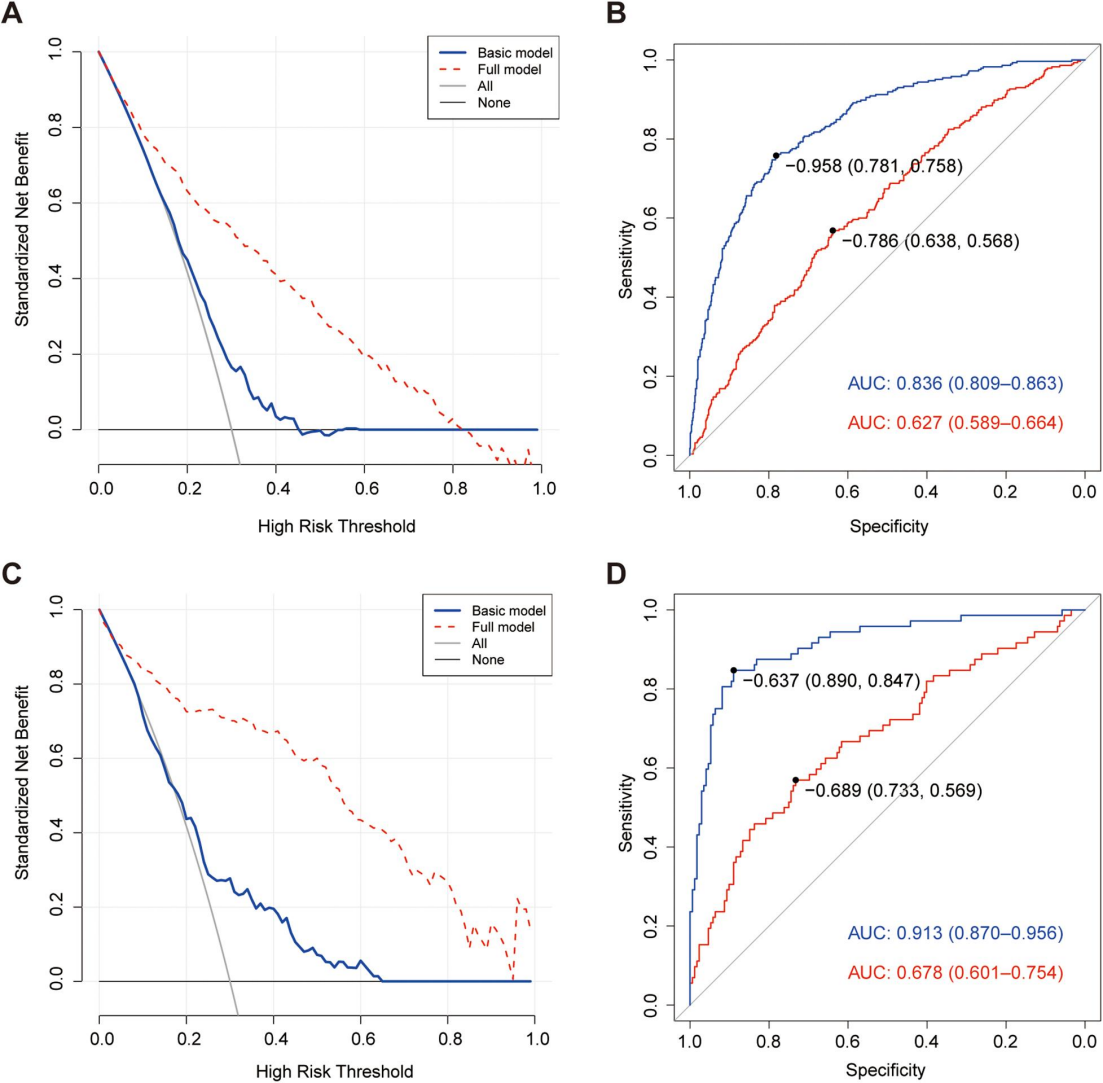
**(A)** Decision curve analysis of the two models in the training set for SAP in children. **(C)** Decision curve analysis of the two models in the cross-validation set for SAP in children. **(A,C)** The solid blue line corresponds to the basic model, and the dotted red line corresponds to the full model. The solid horizontal line without numbers in black represents mild adenovirus pneumonia, while the solid curve in grey represents severe adenovirus pneumonia. The net benefit of the full model was greater than that of the basic model when the significant factors were added. **(B)** ROC curve analysis of the two models in the training set for SAP in children. The solid red line corresponds to the basic model, and the blue solid line corresponds to the full model. The basic model includes age, sex, the number of days the patient was hospitalized, influenza infection status, bacterial infection status, IgA status, IgM status, and percentages of CD8^+^ T lymphocytes [CD8^+^ (%)] and CD16^+^/56^+^ status (%). The full model includes both the factors in the basic model and the seven significant factors namely. MP infection, complement components 3 (C3 and C4), IgG, CD3^+^ (%), CD4^+^ (%) and CD19^+^ (%). **(D)** ROC curve analysis of the two models in the cross-validation set for SAP in children. The solid red line corresponds to the basic model, and the solid blue line corresponds to the full model. The basic model includes age, sex, the number of days the patient was hospitalized, influenza infection, bacterial infection, MP infection, multiple infections, IgM, CD8^+^ (%) and CD16^+^/56^+^ (%). The full model includes both factors in the basic model and the seven significant factors namely, C3, C4, IgA, IgG, CD3^+^ (%), CD4^+^ (%) and CD19^+^ (%).

Decision curve analysis (DCA) further demonstrated a clear net clinical benefit when the significant factors identified above were added to the basic model, as shown in Figure 2A for the training set and Figure 2C for the cross-validation set. In terms of discriminative ability, including these factors improved the area under the receiver operating characteristic curve (AUC) from 0.627 to 0.836 in the training set (Figure 2B), representing a 22.9 percentage point increase, with the DeLong test confirming a statistically significant difference (*Z* = −10.647; *P* < 0.001). Similarly, in the cross-validation set, the AUC improved from 0.678 to 0.913 (Figure 2D), and the difference was also statistically significant (*Z* = −6.135; *P* < 0.001). These results collectively confirm that the inclusion of significant factors provides a substantial and statistically meaningful improvement in SAP prediction.

### 3.4 Establishment of the prediction nomogram model

**Figure 3:**
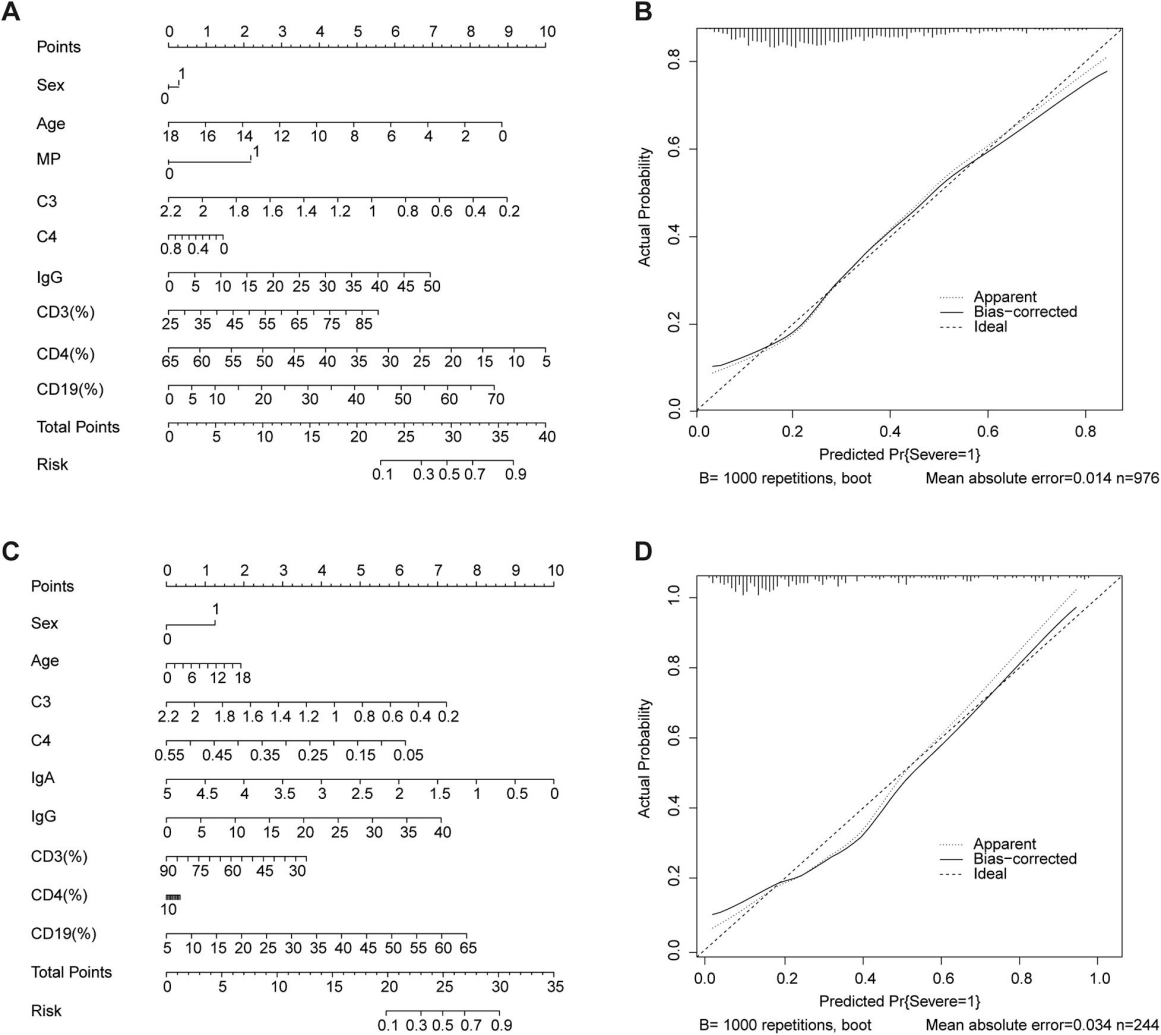
**(A)** Prediction nomogram of significant factors in the training set for SAP in children. Specifically, there is a reference line at the top, which is used to read the score points from all the factors in the regression model. The total score is calculated by summing the scores corresponding to each predicted point. The higher the total score is, the greater the risk that patients with AVP will progress to SAP. **(B)** Calibration curve of the nomogram model in the cross-validation set for predicting SAP in children. **(C)** Prediction nomogram of significant factors in the training set for SAP in children. Specifically, there is a reference line at the top, which is used to read the score points from all the factors in the regression model. The total score is calculated by summing the scores corresponding to each predicted point. The higher the total score is, the greater the risk that patients with AVP will progress to SAP. **(D)** Calibration curve of the nomogram model in the cross-validation set for predicting SAP in children. MP, mycoplasma infection; C3, complement component 3; C4, complement component 4; IgG, immunoglobulin G; IgA, immunoglobulin A; CD3 (%),percentage of T lymphocytes; CD4 (%), percentage of CD4^+^ T lymphocytes; CD19 (%), percentage of CD19^+^ T lymphocytes.

To further evaluate the combined contribution of the identified significant independent factors, we constructed a predictive nomogram (Figure 3A). In the training set, the discriminative ability of the nomogram was good, with a C-index of 0.731. The model also exhibited high sensitivity (0.924), moderate accuracy (0.787) and specificity (0.456) in this cohort, indicating its effectiveness in identifying severe cases.

Calibration of the nomogram was assessed using a bootstrap method with 1000 resamples. The calibration curve for the training set (Figure 3B) was used to plot the predicted probability of SAP against the actual observed frequency. As shown in the figure, the *x*-axis represents the predicted probability of the SAP, whereas the *y*-axis represents the actual observed probability. The diagonal dotted line indicates complete calibration; that is, the predicted probability is completely consistent with the actual probability. The close alignment of the calibration curve with the diagonal reference line indicates a high level of agreement between the predicted and observed outcomes, confirming the model's reliability in risk estimation.

The nomogram was further validated in the independent cross-validation set (Figure 3C), where it achieved a C-index of 0.812, reflecting strong and consistent discriminative performance. In this validation cohort, the model maintained high sensitivity (0.914) and exhibited improved specificity (0.619) and overall accuracy (0.826). The corresponding calibration curve (Figure 3D) also demonstrated excellent calibration, with predicted probabilities closely matching observed event rates across the risk spectrum.

These results collectively indicate that the nomogram is well-calibrated and exhibits robust discriminative ability across both training and validation datasets, supporting its potential utility for individualized risk stratification in clinical practice.

## 4 Discussion

HAdV infection is among the most common causes of pneumonia in children and accounts for a substantial proportion of community-acquired lower respiratory tract infections in children under five years of age (23, 24). When adenovirus progresses to severe adenovirus pneumonia (SAP), multiple system dysfunctions, including respiratory, circulatory, digestive, and nervous system complications, can frequently occur, leading to long-term pulmonary sequelae in a subset of affected children (25, 26). This high morbidity, high mortality, and long-term disability impose substantial economic and psychological burdens on both families and society.

The objective of this study was to investigate the predictive value of laboratory parameters related to humoral and cellular immunity for SAP in children in a clinical setting. Complement components C3 and C4 are the core effector molecules of the complement system, bridging the gap between innate immunity and adaptive immunity, and are essential for resistance to microbial infections and removing immune complexes and damaged cells (27). The reduced serum C3 and C4 levels indicate systemic complement consumption, reflecting a state of hyperinflammation characteristic of severe disease. This pathophysiological insight positions the complement cascade as a potential therapeutic target, suggesting that interventions such as complement inhibitors can help modulate the excessive immune response. IgG exerts antiviral effects through neutralization, conditioning, and antibody-dependent cell-mediated cytotoxicity. With respect to nonenveloped viruses, IgG can mediate intracellular neutralization in cells, and block the fusion of the virus with the host cell, thereby inhibiting virus entry and spread. For patients with primary or secondary low IgG levels, intravenous immunoglobulin supplementation can quickly restore neutralizing antibody levels and reduce the incidence of severe cases (28). Factors such as age and sex may influence SAP occurrence in children; these factors were considered in the present study (7, 29, 30). Through retrospective analysis of 1220 hospitalized children with AVP, we identified seven independent factors significantly associated with SAP compared with mild cases in children after statistical adjustment. These factors included MP infection, complement components (C3 and C4), IgG, and the relative counts of CD3^+^ and CD4^+^ T lymphocytes and B lymphocytes [CD19^+^ (%)].

Similarly, previous research has suggested that MP coinfection is a significant risk factor (OR = 5.0), which corroborates our result (OR = 1.372), albeit with different effect sizes possibly due to differences in population or viral serotype distribution (31).MP coinfection acts as a critical immune modulator by triggering a hyperinflammatory cascade and disrupting immune homeostasis. Mechanistically, MP can induce the secretion of proinflammatory cytokines and chemokines (32). Clinically, this coinfection status serves as a molecular indicator of enhanced immune dysregulation, and targeted strategies such as early macrolide administration, including azithromycin, may mitigate this synergistic inflammatory effect by inhibiting viral replication and modulating macrophage activation (32).

CD3^+^ T lymphocytes are central mediators of cellular immunity, and CD4^+^ T lymphocytes coordinate innate and adaptive immune responses. In chronic viral infection or in the tumor microenvironment, continuous antigen stimulation can cause CD4^+^ T cells to enter an exhausted state, resulting in blocked virus clearance (33). B lymphocytes are precursors of antibody-secreting plasma cells linking antigen presentation to humoral immunity. They recognize viral antigens through surface immunoglobulins and subsequently clone, amplify, and differentiate into plasma cells to produce specific antibodies. A decrease in B lymphocytes indicates impairment of antibody production and increased risk of severe infection (34). The role of B cells is in contrast with that in the literature, suggesting that complex involvement beyond antibody production merits further subset analysis. For example, B cell proportions were reported to be elevated and T/NK cell proportions to be lower in patients with SAP, which is in agreement with our findings of altered CD3^+^ and CD19^+^ percentages (35). Moreover, the significant decrease in the percentages of CD3^+^ and CD4^+^ T lymphocytes in patients with SAP echoes the pronounced lymphopenia described for HAdV-7 infections in a previous study (36).

Given that the clinical progression of adenovirus pneumonia in children is a multistep and multifactorial process, it is unlikely that any single factor plays a dominant role. Prediction models incorporating multiple attributes are more valuable than models reporting individual factors alone, as the contribution of one attribute may be enhanced or masked by the presence of another (37, 38). To address this issue, we developed a predictive nomogram based on factors that are weakly correlated, independent of demographic covariates, and significantly associated with SAP in children. This model demonstrated favourable predictive performance across multiple dimensions. The nomogram exhibited good predictive ability and accuracy. Several studies have attempted to predict disease severity and sequelae of pediatric adenovirus pneumonia using nomogram techniques (39, 40). However, the factors incorporated in these models are inconsistent, likely because of differences in patient characteristics, statistical power, and potential residual confounding. The key novelty of this study lies in the synthesis of these clinical and immunological predictors into a validated, integrated risk stratification tool. Studies have successfully identified independent risk factors, such as high IgE, LDH, and CRP levels (6, 35). Our study constructed and internally validated a predictive nomogram for quantifying the individual risk of SAP by integrating key clinical and immune variables, including age, sex, MP coinfection status, complement components C3 and C4, IgG, and lymphocyte subsets, thereby advancing research in this field. This integrated approach not only enhances individualized risk stratification, but also proposes a unified pathophysiological framework for SAP, which is characterized by the simultaneous excessive activation of innate immunity, humoral immune disorders, and impaired cellular defence. By linking the predictors of the nomogram to these potential immune mechanisms, our model provides clinically operable tools and potential molecular targets for future immune regulation strategies.

In addition to its apparent strengths, such as its large sample size, successful internal validation and satisfactory calibration and discrimination performance, several potential limitations of this study should be acknowledged. First, because of its retrospective design based on routine clinical examination, we were unable to assess the levels of established proinflammatory cytokines that are known to increase because of severe disease. If these indicators are included, it may provide a more comprehensive understanding of the inflammatory state. Therefore, prospective studies should be performed in the future to evaluate the cellular/humoral immune parameters studied here and a wider range of cytokine combinations to comprehensively determine disease severity to predict immune characteristics. Second, owing to the limitation of examination during hospitalization, we diagnosed only children infected with adenovirus without distinguishing specific serotypes; thus, observing the changes in clinical characteristics and factors influencing SAP in detail according to serotypes was impossible. Third, immunological parameters were measured only at the initial postadmissiontime point, precluding the assessment of their dynamic changes. Future studies incorporating longitudinal follow-up data should investigate how temporal trends in these markers correlate with clinical outcomes, thereby enabling more refined risk stratification. Finally, a limitation of this study is its single-center design, which may affect the generalizability of our results. In response to this concern, we conducted internal validation by randomly splitting our queues. This process proves the consistent identification of significant predictors, indicating that the nomogram does not overfit our specific datasets. Although the current internal validation is reassuring, internal validation cannot replace external validation. External validation in multicenter or publicly published cohorts is still necessary to confirm the clinical utility of the nomogram.

## 5 Conclusion

In summary, our findings reveal seven significant factors for SAP development in children, including six immunology-related laboratory parameters. More importantly, a nomogram prediction model based on these factors can effectively identify children with adenovirus pneumonia who are at a high risk of disease progression, thereby providing a strong tool to support clinical decision-making. Although further validation through prospective studies is warranted, our initial internal validation confirms the ability of the model to perform rapid risk assessment at admission on the basis of these immunological markers, enabling timely identification of pediatric patients at increased risk of developing SAP.

## Data Availability

The original contributions presented in the study are included in the article/Supplementary Material, further inquiries can be directed to the corresponding author.
